# Treatment of Intracranial Aneurysms With the SILK Embolization Device in a Multicenter Study. A Retrospective Data Analysis

**DOI:** 10.1093/neuros/nyw123

**Published:** 2017-01-28

**Authors:** José Manuel Pumar, Alexandra Banguero, Hugo Cuellar, Leopoldo Guimaraens, Javier Masso, Salvador Miralbes, Miguel Blanco-Ulla, Fernando Vazquez-Herrero, Miguel Souto, Miguel Gelabert-Gonzalez

**Affiliations:** * Department of Neuroradiology, Hospital Clinico Universitario, Universidad de Santiago de Compostela, Travesia de la Choupana s/n, Santiago de Compostela, Spain; ‡ Department of Neurosurgery and Radiology at LSU Health Sciences Center, Shreveport, Louisiana; § Department of Interventional Neuroradiology, Hospital General de Cataluña, San Cugat, Barcelona, Spain; ¶ Department of Neuroradiology, Hospital Universitario de Donostia, San Sebastian, Spain; || Department of Neuroradiology, Hospital Universitario Son Espases, Palma de Mallorca, Spain

**Keywords:** Flow-diverter Embolization device, Intracranial aneurysm, Stent embolization, SILK

## Abstract

**BACKGROUND:**

Flow-diverter technology has become an important stent-based embolization tool in the treatment of complex cerebrovascular pathology. We report here the experience of 4 Spanish centers with using the SILK flow-diverter (SFD) device.

**OBJECTIVE:**

To evaluate the safety and efficacy of using the SFD in the endovascular treatment of intracranial aneurysms with complex morphology.

**METHODS:**

We retrospectively examined a prospectively maintained database of patients treated with SFD devices between July 2008 and December 2013 at 1 of 4 institutions in Spain. Data regarding patient demographics, aneurysm characteristics, and technical procedure were analyzed. Angiographic and clinical findings were recorded during the procedure and at 12 months postoperatively.

**RESULTS:**

A total of 175 SFD devices were implanted in 157 patients (women/men: 119/38; mean, median, and range of age: 56.2, 56.7, and 19-80 years, respectively), who were treated in a delayed manner (3-6 months from the event) for 180 aneurysms (165 unruptured and 15 ruptured). Adverse events (acute and delayed) were observed in 28.7% of cases (45/157), and most were resolved (19.1%; 30/157). Six months after the procedure, total morbidity and mortality were 9.6% (15/157) and 3.2% (5/157), respectively. Long-term imaging follow-up showed complete occlusion, neck remnants, and residual aneurysm in 78.1% (100/128), 14.0% (18/128), and 7.8% (10/128) of cases, respectively.

**CONCLUSIONS:**

The SFD device is an effective tool for the treatment of challenging aneurysms, and allows complete occlusion within a year of the procedure in most patients, with morbidity and mortality comparable to those previously reported for similar devices.

**ABBREVIATIONS**
RADAR: Retrospective Analysis of Delayed Aneurysm Ruptures
SFD: SILK flow diverter
SAH: subarachnoid hemorrhage

**T**he advent of flow-diverter technology has provided a new therapeutic option for managing large wide-neck aneurysms. The flow-diverter device modifies blood flow within and around the aneurysm inflow zone by means of a stent, which eventually leads to thrombosis inside the aneurysm and subsequent contraction of the aneurysmal sac, while flow into the parent vessel and perforating branches is preserved. The present report provides a detailed overview of the SILK flow-diverter (SFD) embolization device, including an overview of its mechanism of action and deployment technique, and further describes an assessment of its safety and efficacy in a consecutive series of patients with intracranial aneurysm.

## PATIENTS AND METHODS

### Patient Population

The study involved a retrospective review of multicenter data regarding a consecutive series of patients with intracranial aneurysms, treated with the SFD between July 2008 and December 2013 at 1 of 4 institutions. Our ethic board approved this study. Patient selection for endovascular treatment was performed by a multidisciplinary team of interventional neuroradiologists, neurologists, and neurosurgeons. The treatment decision was based on the size, location, and morphology of the aneurysm, and the clinical status of the patient. The patients and their relatives were informed regarding the complications associated with the diagnosed condition, the treatment options available, as well as the risks. Endovascular treatment was performed only after informed consent was obtained.

### Therapeutic Strategy

All patients received clopidogrel (75 mg/day) and aspirin (150 mg/day) for at least 7 days before the procedure. Tests for responsiveness to clopidogrel and aspirin were not available at all hospitals. In some cases, a loading dose of 300 to 600 mg clopidogrel and 300 mg oral aspirin or 0.5 to 1 g intravenous aspirin were used as an alternative.

All procedures were performed after inducing general anesthesia and applying therapeutic heparinization with activated clotting times of approximately 300 s. After the procedure, heparin was continued for at least 24 h, whereas dual antiplatelet medication, including clopidogrel (75 mg/day) and aspirin (150 mg/day), was continued for at least 6 months. After this period, if follow-up imaging investigations revealed no stenosis, clopidogrel was stopped, while aspirin (150 mg/day) was continued permanently; if imaging revealed stenosis, dual antiplatelet therapy was continued.

### Endovascular Procedure

The SFD deployment strategy aimed for strict compliance with the recommendations of the SFD device manufacturer (Balt Extrusion, Montmorency, France). The choice of stent length and diameter was based on pre- and intraprocedural imaging investigations. The stent length was chosen such that the stent extended, at least 10 mm, beyond both sides of the aneurysm neck, while the stent diameter was approximately the diameter of the parent artery. When there was a large discrepancy between the diameters of the proximal and distal ends of the parent vessel, the choice was based on the diameter of the proximal part. The stent was never oversized, and adequate openness was obtained. The stent was slowly deployed from the microcatheter, and secured against the vessel wall by pushing the microcatheter distally through the stent.

Deployment failure was evaluated in terms of the following aspects: failure to advance a long SFD through the delivery catheter; poor SFD opening on deployment; poor SFD positioning; stent displacement; and vascular tortuosity.

Angiography was performed immediately after the procedure, and at 6-month and 1-year follow-ups, in order to assess the degree of occlusion of the aneurysm. Aneurysm occlusion grade was established using the Montreal grading system.

### Clinical Complications and Related Morbidity and Mortality

Periprocedural and postoperative complications were evaluated. Clinical outcome was evaluated at discharge and at the 6-month follow-up using the modified Rankin scale. The primary outcomes assessed were neurological morbidity and mortality. Neurological morbidity was defined as the composite of the following neurological complications: spontaneous aneurysm rupture, ipsilateral intracranial hemorrhage, ischemic stroke, stenosis of the parent artery, and cranial neuropathy. All complications were retrospectively revised by 3 senior investigators that determined the category of the event as “major” or “minor,” with “major” defined as an ongoing clinical deficit at 7 days after the event. All major adverse events were considered when evaluating the overall incidence of neurological morbidity and mortality.

Acute and subacute complications (occurring within 2 weeks of the procedure), as well as delayed complications (occurring between 2 weeks and 6 months after the procedure) were classified according to a protocol similar to that employed by Berge et al.^1^

### Data Collection and Literature Review

Descriptive data are presented as mean ± standard deviation of the number and percentage of analyzed cases. In order to compare our results with those reported in the past, relevant databases (Pubmed, Scopus, Google Scholar, EMBASE via Ovid, and Web of Science) were searched using the keywords “intracranial aneurysms,” “Silk flow diverter,” and “Pipeline + Silk flow diverters” (accessed April 2015). The bibliographic sections of the identified studies were searched for relevant literature published between January 2005 and April 2015. The studies with the following characteristics were included in the analysis: publication language, English; number of patients included, >10 patients; treatment using Silk or Pipeline devices; and data provided regarding postoperative complications and aneurysmal occlusion rates. Case reports, review articles, and technical notes were not considered.

## RESULTS

### Patient and Aneurysm Characteristics

A total of 157 patients (119 women, 75.7%; 38 men, 24.2%) were treated during the analyzed period for 180 aneurysms. The range, mean, and median of the age in this consecutive series of patients were 19 to 80, 56.2, and 56.7 years, respectively.

A total of 80 patients (51.1%) were asymptomatic, whereas 77 (49.5%) were symptomatic; specifically, 31 patients (19.7%) underwent a diagnostic imaging test for nonspecific symptoms such as headache or dizziness, 25 patients (15.9%) had cranial nerve deficit, 4 had ischemic stroke, and 2 had transient ischemic events. A total of 15 patients presented with recurrent aneurysms and subarachnoid hemorrhage (SAH), and were treated for an average time of 160 days after SAH was diagnosed (range, 90-180 days).

Most aneurysms (92.2%) were located in the anterior territory (158, internal carotid artery; 6, middle cerebral artery; and 2, anterior cerebral artery), while the remainder (7.7%) were located in the posterior territory (8, basilar artery; 4, vertebral artery; and 2, posterior cerebral artery). In terms of aneurysm morphology, 166 of 180 (92.2%) aneurysms were saccular, and 14 of 180 (7.8%) were fusiform. The mean aneurysm size was 11.4 mm (median, 11.5 mm; range, 2-42 mm).

A total of 128 aneurysms (71.1%) had an unfavorable dome-to-neck ratio (<1.6), with a mean dome-to-neck ratio of 1.5.

### Intraprocedural Difficulties

A total of 175 SFD devices were deployed in 157 patients with 180 aneurysms. Of the deployed devices, 6.3% (11/175) showed incorrect deployment on angiography. Folding/kinking or inadequate opening represented the cause for incorrect deployment of 8 stents, which led to acute thrombosis of the system in 4 cases (all in patients with very tortuous anatomy at the deployment site, which prevented the proper opening of the SFD, resulting in thrombosis). Additional procedures such as balloon angioplasty were required in 6 patients. In 2 cases with acute in-stent thrombosis and previous balloon test occlusion, we elected to sacrifice the parent vessel in order to avoid the risk of distal emboli. In 2 other cases, the devices were displaced, implying inadequate or incomplete coverage of the aneurysmal neck; one of these cases was resolved with the introduction of a new SFD stent, performed during a second intervention; in the other case, the device was found to be frayed, and it was decided to deploy a second SFD stent within the previously implanted SFD stent.

The majority of patients were treated with a single SFD device (84.1%), but 10 patients were treated with 2 stents to cover the entire length of the aneurysm and the dysplastic portion of the parent artery. In 15 further cases, other devices were used as complements to SFD treatment; additional coiling was performed in 10 patients, while 5 additional Leo stents were implanted in 5 patients prior to SFD placement, to allow anchoring of the SFD stent, with no evidence of immediate thrombosis of the system. We typically placed a Leo stent as a bridge to support the SFD device and compensate for its low radial force, which enabled to avoid herniation into a large/giant circumferential aneurysm.

### Clinical Complications and Related Morbidity and Mortality

The rate of acute and subacute morbidity was 7.6% (12/157 patients), while the mortality rate was 3.2% (5/157 patients). Three deaths were due to a large ipsilateral ischemic stroke following the procedure of treating aneurysms of the basilar artery (2 cases) and of the supraclinoid segment of the internal carotid artery (1 case), with complete occlusion of the SFD stent and parent artery at 2 to 5 days after the procedure. The other 2 patients who died had delayed rupture of giant paraophthalmic aneurysms; death occurred at 1 and 6 days after the procedure, and was due to SAH and large ipsilateral intracranial hemorrhage; in both cases, postmortem examination showed massive organizing intraluminal thrombus and wall thinning at the site of the rupture, with mural necrosis.

Delayed complications (ie, occurring between 2 weeks and 6 months after the procedure) included morbidity in 2.0% of cases (3/152 patients). In the present series, there were no patients with intracerebral bleeding or procedure-related death occurring later than 1 week after the procedure.

Three patients had hemorrhagic complications considered to be procedure-related (2, hematomas next to the aneurysm; 1, SAH). Ischemic events occurred in 13 patients, and 4 were considered to be procedure-related, while 9 were considered to be device-related (3, side branch occlusion; and 6, in-stent thrombosis).

There were a total of 45 adverse events (28.7%, 45/157), of which 30 (19.1%, 30/157) were minor events that resolved in less than 7 days, whereas 15 (9.6%, 15/157) were considered major adverse events consisting of 10 cases with permanent deficits (6.3%, 10/157) and 5 deaths (3.2%, 5/157). There was no significant difference regarding morbidity and mortality between patients with SAH and those with unruptured aneurysms. The group of patients with anterior circulation aneurysms showed morbidity and mortality rates of 8.8% and 1.8%, respectively. The group of patients with posterior circulation aneurysms showed morbidity and mortality rates of 0.7% and 14.3%, respectively. Overall, the 6-month morbidity and mortality rates were 9.6% and 3.2%, respectively (Table 1).

**TABLE 1.** Clinical Complications Outcomes

		Time of occurrence		
	Number of occurrences	Procedure	1-15 days	15 days-6 months
Minor complications	30			
Allergic reactions	8 (5.1%)	0 (0%)	8 (5.1%)	0 (0%)
Insertion site hematoma/bleeding	6 (3.8%)	6 (3.8%)	0 (0%)	0 (0%)
Arterial dissection	3 (1.9%)	3 (1.9%)	0 (0%)	0 (0%)
Retroperitoneal hematoma	5 (3.2%)	6 (3.9%)	0 (0%)	
Transient Ischemic Attack	8 (5.1%)	0 (0%)	6 (3.8%)	2 (1.3%)
Major complications	15			
Spontaneous rupture	0 (0%)	0 (0%)	0(0%)	0 (0%)
Intracranial hemorrhage	2 (1.3%)	0 (0%)	2 (1.3%)	0 (0%)
Ischemic stroke	5 (3.2%)	0 (0%)	3 (1.9%)	2 (1.3%)
Parent artery stenosis	8(4.5%)	7(3.8%)		1 (0.7%)
Cranial neuropathy	0 (0%)	0 (0%)	0 (0%)	0 (0%)
Neurological morbidity	15 (9.6%)		12 (7.6%)	3 (2.0%)
Neurological mortality	5 (3.2%)		5 (3.2%)	0 (0.0%)

### Occlusion Rate

Immediate postprocedural angiography indicated no complete occlusions (grade III), 22 (12.2%) grade II occlusions, 116 (64.4%) grade I occlusions, and 42 (23.4%) cases with no significant change in aneurysmal filling.

Our follow-up protocol included angiographic investigations at 6 and 12 months after the procedure. However, because the actual timing varied among the patients in our case series, we decided to include in our analysis only the angiographic data obtained at the 12-month follow-up visit, as such data seemed the most reliable. Such angiographic follow-up data was available for 121 patients with 128 aneurysms. At the 1-year follow-up, complete occlusion (class 1, as described by Roy et al^2^) was noted in 100 of 128 aneurysms (78.1%). Residual aneurysm neck and recurrent aneurysm (class 2/3) were noted in 21.8% of cases (28/128), with residual necks accounting for 14% (18/128 aneurysms), and recurrent aneurysm accounting for 7.8% (10/128 aneurysms) of cases, respectively.

As for the location of the residual aneurysms, the majority of the partial occlusions were noted in the ophthalmic/paraophthalmic (10/28, 35.7%) and cavernous (7/28, 25%) segments of the internal carotid artery; fewer residual aneurysms (5/28, 17.8%) were noted in the posterior communicating segment and posterior cerebral artery, and only 2 posterior circulation aneurysms were located in the basilar artery (2/28, 7.1%). In relation to the size of the residual aneurysms, 60.7% of the partial occlusions corresponded to large aneurysms (10-24 mm), and most were saccular. Of the 28 residual aneurysms, 7 (25%) had previously undergone unsuccessful endovascular treatment.

Of the 128 patients, 8 (6.3%) showed intrastent stenosis of <50% of the vascular lumen, with parental artery patency and no flow limitation within a year after the procedure. In 3 cases, stenosis was resolved spontaneously after resuming clopidogrel treatment.

## DISCUSSION

This study was a retrospective review of a consecutive series of 157 patients admitted to 4 Spanish hospitals for treatment of 180 intracranial aneurysms via endovascular implantation of 175 SFD devices. The results of the present series are further discussed in the context of the results reported in the literature regarding the outcome of treatment using flow-diverter stents (Table 2).

**TABLE 2.** Studies Included

Publication author/year	Multicenter/single center/study type	Total patients	N° aneurysms/SFD
Briganti et al, 2012^3^	Multicenter/Italy—retrospective	273	295/152 SILK-183 Pipeline
Velioglu et al, 2012^4^	Single center/Istanbul retrospective	76	87/73
Berge et al, 2012^1^	Multicenter/France—retrospective	65	77/73
Byrne et al, 2010^5^	Multicenter/USA/CE—prospective	70	70/70
Piano et al, 2013^6^	Single center/Milan—retrospective	101	104/47 SILK- 57 Pipeline
Lubicz et al, 2010^7^	Multicenter/Belgium—prospective	29	34/35
Maimon et al, 2012^8^	Single center/TelAviv—retrospective	28	32/31
Shankar et al, 2013^9^	Single center/Halifax, Ca—retrospective	19	29/21
Leonardi et al, 2011^10^	Single center/Bolonia—retrospective	25	25/25
Tähtinen et al, 2011 ^11^	Single center/Tampere, Finland—retrospective	24	24/22
Pistocchi et al, 2011^12^	Single center/Paris—prospective	26	30/25 SILK - 7 Pipeline
Wagner et al, 2012^13^	Single center/Copenhague—prospective	22	26/23
Kulcsar et al, 2010^14^	Multicenter/Germany–Switzerland	12	12/13

### Technical Limitations

Treatment with the SFD system presents specific problems, mainly related to difficulties in deployment. In this regard, our results are consistent with those reported from the largest case series published by Briganti et al in 2012.^3^ In the rest of the studies regarding technical feasibility, the rate of successful deployment varies between 75% and 96%, with an average of 88.6%.^1,4-13^ A direct comparison between our results and the results reported by these previous studies^3,5^ is not possible because of the variations in key factors such as patient selection criteria and population size. Nevertheless, both multicenter studies and anecdotal publications agree that the deployment of the SFD stent represents the technical bottleneck related to this type of procedure (Table 3). We believe that such difficulties are due to the fact that significant experience is needed to implant flow-diverter devices, as such a procedure is different than that followed for implantation of supporting stents.

**TABLE 3.** Percentages of SILK Deployment Difficulties

Publication author/year	N° SILK	Difficulties of SILK deployment: %
Shankar et al, 2013^9^	21	5%
Briganti et al, 2012^3^	152	4%
Velioglu et al, 2012^4^	91	23%
Berge et al, 2012^1^	73	12%
Byrne et al, 2010^5^	70	21%
Piano et al, 2013^6^	50	4%
Lubicz et al, 2010^7^	35	15%
Maimon et al, 2012^8^	31	4%
Kulcsar et al, 2010^14^	13	8%
Leonardi et al, 2011^10^	25	4%
Tähtinen et al, 2011^11^	22	25%
Pistocchi et al, 2011^12^	25	4%
Wagner et al, 2012^13^	23	18%
Average	48.5	11.3%
Our results	175	6.28%

In the present series including 175 SFD devices implanted in 157 patients, technical difficulties were encountered in 6.3% of cases, implying a technical feasibility of 93.7%. The main risk factor for technical failure was insufficient attention during the preparation, navigation, positioning, and deployment of the SFD device, along with other aspects such as vascular tortuosity and selection of highly complex aneurysms for this type of treatment. Another crucial reason for technical failure was the inability to advance the Vasco microcatheter beyond the aneurysmal neck while ensuring sufficient safety margins for navigating the SFD stent; other authors have also expressed difficulties in navigating the microcatheter.^7^

### Occlusion Rate

At the 12-month follow-up, we observed complete occlusion in 78.1% (100/128) of aneurysms. In line with most published series,^5,15^ most aneurysms were located in the anterior territory, with only 7.7% (14/180) in the posterior territory. Although, in our series, complete occlusion occurred mostly between 6 and 12 months after the procedure, our experience has indicated that it is not possible to predict the progression to permanent thrombosis and contraction of the aneurysmal sac. Specifically, obvious changes in intra-aneurysmal flow can lead to complete occlusion of the aneurysm immediately after stent deployment. However, no such cases were noted in the present series (Table 4). The use of dual antiplatelet therapy for 6 months and sometimes longer increases the uncertainty as to when occlusion will occur. The complete occlusion rate at 1 year after implantation of a Pipeline device was reported at 86.8%, which improved to 93.4% and 95.2% at 3 and 5 years after the procedure, respectively.^16^ Based on these results, we plan to continue our long-term follow-up to 5 years, in an effort to enable comparison of our results against those reported in the clinical trials for the Pipeline device.

**TABLE 4.** Follow-up of Occlusion Grade

		Follow-up/complete occlusion grade	
Publication author/year	N° aneurysms	<6 months	>6 months
Briganti et al, 2012^3^	295	85%	
Velioglu et al, 2012^4^	87		87%
Berge et al, 2012^1^	77	68%	84.5%
Byrne et al, 2010^5^	70	50%	
Piano et al, 2013^6^	47	86%	87%
Lubicz et al, 2010^7^	34	69%	
Maimon et al, 2012^8^	32		70%
Shankar et al, 2013^9^	29		59%
Leonardi et al, 2011^10^	25		60%
Tähtinen et al, 2012^11^	24		70%
Pistocchi et al, 2012^12^	30		79%
Wagner et al, 2012^13^	26	68%	86%
Average	65	71%	76%
Our results	180	71%	78.1%

Recurrence is a major concern in conventional endovascular treatment of aneurysms. In our study, as in previous investigations by Kulcsar et al,^14^ Lubicz et al,^7^ Szikora et al,^17^ and Berge et al,^1^ no recanalization or reduction in occlusion grade was found, indicating that treatment with flow-diverter stents does not lead to worsening of the aneurysm. In the population described by Byrne et al,^5^ only 2 patients showed a reduction in occlusion grade. The absence of recanalization or reduction in occlusion grade noted in our case series represents a very promising result.

### Clinical Complications and Related Morbidity and Mortality

The mortality and morbidity rates noted in our cohort were comparable to those reported by recent studies concerning SFD and Pipeline devices. In the present study, acute and subacute morbidity was 7.6% (12 of 157 patients), and mortality was 3.2% (5 of 157 patients). There are few reports on acute complications after SFD implantation, as most studies report combined rates of acute, subacute, and delayed complications. Berge et al^1^ reported a rate of 7.7% for acute and subacute morbidity, which is comparable to 7.6% noted in the present series, but a mortality rate of 0%, which contrasts with 3.1% noted in the present study. In terms of major complications noted at the 6- and 12-month follow-up, Berge et al^1^ found a permanent morbidity rate of 7.8% and a mortality rate of 3%, which are comparable with the values noted in our study (4.4% and 0%, respectively; Table 5).

**TABLE 5.** Morbidity and Mortality

Publication author/year	N° patients	Permanent morbidity %	Mortality %
Briganti et al, 2012^3^	273	3.7%	5.9%
Velioglu et al, 2012^4^	76	6.6%	6.6%
Berge et al, 2012^1^	65	7.8%	3%
Byrne et al, 2010^5^	70	4%	8%
Piano et al, 2013^6^	101	3%	3%
Lubicz et al, 2010^7^	29	15%	4%
Maimon et al, 2012^8^	28	10.7%	3.6%
Shankar et al, 2013^9^	19	10%	5%
Leonardi et al, 2011^10^	25	4%	8%
Tähtinen et al, 2012^11^	24	4%	4%
Pistocchi et al, 2012^12^	26	3.7%	0%
Wagner et al, 2012^13^	22	5%	5%
Average	63	6.5%	4.7%
Our results	157	4.4%	3.2%

When reviewing the relevant literature, it is difficult to compare between the SFD and the Pipeline devices in terms of the incidence of acute arterial occlusion, mainly due to the heterogeneity of the sample population included in each study. Nonetheless, acute arterial occlusion appears to be somewhat more frequent with SFD.^1^ Specifically, in the present study, acute occlusion was noted in 2.5% of cases, compared to 10% in the series described by Byrne et al,^4^ 8% in the experience of Lubicz et al,^7^ and 2.4% in the series described by Szikora et al.^17^ In comparison, acute arterial occlusion was not achieved using the Pipeline device, as reported by Lylyk et al^15^ and Nelson et al.^18^

The morbidity resulting from occlusion of the system appears to be related to the state of the collateral circulation, although 5 patients in the present study developed late thrombosis of the system while remaining clinically asymptomatic. In short, of the 157 patients, 45 had complications (28.7%), of which 39 were directly related to the procedure (24.8%). Of the 45 complications, 30 were resolved (19.1%) and 15 were not (7. 6%). The rate of complications related to the procedure is within the rate reported in other series (4%-38%).^1,4-8,11,14,15,17-20^

Several studies have documented the occurrence of aneurysm rupture following SFD stent therapy.^21-23^ In a retrospective analysis of patients from 12 hospitals, Kulcsar et al^22^ described 13 cases of delayed hemorrhage following SFD stent monotherapy, with most complications occurring within 3 months of the procedure (10 cases, vs 3 cases with hemorrhage noted between 3 and 5 months after the procedure). In our series, there were 2 patients with delayed intracranial parenchyma hemorrhage following SFD stenting, but the average time to post-treatment failure was 3 days (ranging from 1 to 6 days). Our data are thus more similar to the results of RADAR (Retrospective Analysis of Delayed Aneurysm Ruptures) study,^24^ where the average time to post-treatment rupture was 9 days (range, 3-300 days). Bearing in mind the reports by Kulcsár et al^22^ and Turowski et al,^23^ we hypothesize that such ruptures may occur as a result of the rapid and extensive transformation of the existing thrombus or clot, which induces proteolytic activity leading to degradation of the wall and inflammatory weakening, with microhemorrhages on the inside of the vessel, and subsequent rupture.

In the present study, 8 of the patients (6.2%) had intrastent stenosis, which represents a significantly lower rate compared to those reported in previous series.^1,7,9,12,13^ Moreover, these patients were asymptomatic, and spontaneous disappearance of stenosis occurred in 2 patients after restarting antiplatelet therapy, similar to previously reported effects of prolonged antiplatelet therapy.^15^ Further research is warranted to improve our understanding of the behavior of intrastent stenosis.

### Comparison Between the SFD and Pipeline Devices

Although all flow-diverter devices act on the same basic principles, they are slightly different in terms of their design and deployment technique, which may influence the rate of complications and occlusions. By comparing our results with those reported by clinical trials of Pipeline devices (PUFS, IntrePED, and ASPIRE),^25,26^ totaling 1091 patients, we noted that our cohort treated with SFD showed an increased rate of complications. Specifically, our results indicate a significantly higher total rate of neurological morbidity and mortality for the SFD (12.7% vs 7.8% for the combined analysis of the Pipeline clinical trials). Nevertheless, these results may not be comparable, since only one of the trials had a retrospective design similar to ours (IntrePED), whereas the other 2 trials were prospective (PUFS and APIRE). Moreover, these trials included a heterogeneous sample of patients and aneurysms. In addition to these differences, we believe that part of the discordance between our results and the results reported in the Pipeline trials is related to the fact that the SFD system may be more thrombogenic and have less radial force than the Pipeline device, which may increase the need for an adjuvant stent as a construct element, increasing the risk of in- and post-treatment complications such as emboli, flow-diverter thrombosis, and stenosis.

## CONCLUSION

This study represents the largest case series evaluating the use of the SFD system. We present evidence that SFD use is a safe and effective option for the endovascular treatment of wide-neck intracranial aneurysms, and is associated with an acceptable rate of neurological complications.

### Disclosure

The authors have no personal, financial, or institutional interest in any of the drugs, materials, or devices described in this article.

## COMMENT

**F**low-diverting stents have dramatically changed the way many centers manage intracranial aneurysms. The principle of endoluminal reconstruction is 2-fold: first, redirecting blood flow away from the aneurysm and second, providing a scaffold for eventual endothelialization. A number of flow diverters have been developed; and in this report, the authors analyzed an institutional series of 175 Silk flow diverters (SFD) placed in 157 patients over the course of nearly 6 years through a prospectively maintained database. A total of 69.4% of patients achieved 6-12 months of clinical follow-up, with occlusion rates of up to 78%. The authors further analyzed their complication rates and compared these with those of an extensive literature review.

The authors’ SFD series represents the largest thus far reported and demonstrates that the SFD is a safe and effective means for treating intracranial aneurysms. Although the overall conclusions that the SFD device is safe and effective hold, comparison with other devices demonstrates that differential safety may be an issue. As the authors note, there remain technical issues related to deployment of the SFD as well as less radial force compared to, for instance, the Pipeline embolization device (PED), that may make the system more thrombogenic and, therefore, prone to complications. Certainly, this device represents a step forward in aneurysm treatment that will continue to evolve as engineers refine the principles and implementation of flow diversion. The authors should be applauded for carefully evaluating and reporting on such a large cohort of patients to help further our understanding of flow diversion.

**Jason M. Davies**

**Adnan H. Siddiqui**

**Elad I. Levy**

*Buffalo, New York*
